# Procalcitonin as a Potential Prognostic Marker in Advanced Hodgkin Lymphoma: A Case-Based Perspective

**DOI:** 10.7759/cureus.98476

**Published:** 2025-12-04

**Authors:** Mohamad Mosi, Refat Jayyusi, Shaila Saaki, Moin Makrani, Mazin Saadaldin

**Affiliations:** 1 Internal Medicine, Texas Tech University Health Sciences Center, Amarillo, USA

**Keywords:** b lymphocytes, hodgkin lymphoma (hl), lymph node biopsy, procalcitonin (pct), reed–sternberg (hrs) cells

## Abstract

This case report examines the potential role of procalcitonin (PCT) as a prognostic biomarker in classic Hodgkin lymphoma (cHL), focusing on a 61-year-old male with advanced disease and systemic inflammation. The patient presented with progressive dyspnea, bilateral pleural effusions, and mediastinal lymphadenopathy. Imaging revealed hepatic, splenic, and adrenal involvement, consistent with stage IV HL. Axillary lymph node biopsy confirmed lymphocyte-rich cHL, with immunohistochemical markers including CD30, CD15, PAX5, Ki-67, MUM1, and BCL6. Despite broad-spectrum antimicrobial therapy and a comprehensive infectious workup, the patient exhibited rising PCT levels (peak 4.5 ng/mL) and declining albumin levels, with no microbiological evidence of infection. This persistent elevation of PCT in the absence of confirmed infection suggests a possible link between HL-associated inflammation and PCT production, potentially mediated by cytokine-driven paraneoplastic processes. The patient’s clinical deterioration and eventual death underscore the aggressive nature of extranodal HL and raise questions about the utility of PCT as a surrogate marker of disease activity. While PCT is traditionally used to identify bacterial infections, its elevation in this case may reflect tumor-induced systemic inflammation. This observation supports further investigation into the prognostic relevance of PCT in HL, particularly in older patients with extensive disease burden. Clinicians should interpret elevated PCT levels cautiously in oncologic settings and consider malignancy-related inflammation as a contributing factor. Future studies are needed to evaluate PCT dynamics across HL subtypes and treatment phases, potentially integrating it into multimodal prognostic models. This report suggests that PCT may offer valuable insights into disease progression and inflammatory status in HL.

## Introduction

Hodgkin lymphomas (HLs) are malignant lymphoid neoplasms originating from B lymphocytes, most commonly involving the cervical lymph nodes. A hallmark of HL is the presence of large, atypical B cells known as Hodgkin and Reed-Sternberg cells, which play a central role in disease pathogenesis [1].

Lymphoma constitutes about 4% of all newly diagnosed cancer cases and 3.3% of cancer-related deaths in 2024 in the United States [2]. Classic HL (cHL) makes up about 10-15% of all lymphomas. The overall incidence of HL is rare, with roughly 2.6 cases per 100,000 people in the United States [3]. Numerous prognostic factors and their relationship with HL have been studied, such as age, gender, albumin, B symptoms, anemia, etc. [4].

While extensive research has examined the prognostic implications of these clinical and laboratory parameters, few studies have investigated the role of procalcitonin (PCT) in HL. PCT, a protein of 116 amino acids produced by C cells of the thyroid. PCT increases not only with bacterial infection but also with other inflammatory states, such as HL. During systemic inflammation, particularly bacterial infections, PCT levels rise significantly. This elevation is mediated by inflammatory cytokines such as interleukin-6 (IL-6) and tumor necrosis factor-alpha (TNF-α), which stimulate PCT production from neuroendocrine cells located in the lungs and intestine [5].

This case explores the potential relationship between PCT levels and HL, with a particular focus on evaluating PCT as a prognostic inflammatory biomarker. Understanding this association may offer insights into disease activity, treatment response, and overall prognosis in patients with HL.

## Case presentation

A 61-year-old male with a significant smoking history, chronic obstructive pulmonary disease, and heart failure with reduced ejection fraction presented to the emergency department with progressive dyspnea that began two months prior and acutely worsened over the preceding three days. The patient reported a productive cough with scant white sputum. On initial evaluation, vital signs revealed hypotension (BP 100/60 mmHg), tachycardia (HR 100 bpm), hypoxemia (SpO₂ 85% on room air), and normothermia (36.9°C). Physical examination demonstrated decreased bilateral breath sounds, more pronounced on the left, with diffuse wheezing. The patient’s admission laboratory results are summarized in the table below.

**Table 1:** Admission laboratory results WBC: white blood cell count, Hg: hemoglobin, Hct: hematocrit, PLT: platelets, TP: total protein, LDH: lactate dehydrogenase, ALP: alkaline phosphatase, ALT: alanine aminotransferase, AST: aspartate aminotransferase, TB: total bilirubin, BUN: blood urea nitrogen, Cr: creatinine, Na: sodium, K: potassium, Ca: calcium, Mg: magnesium, HDL: high-density lipoprotein, TG: triglycerides, CRP: C-reactive protein, PCT: procalcitonin, ABG: arterial blood gas, PCO₂: partial pressure of carbon dioxide, PO₂: partial pressure of oxygen, HCO₃: bicarbonate ion

Parameter	Patient value	Reference range
WBC	10.2 ×10⁹/L	4.0-11.0 ×10⁹/L
Hg	11 g/dL	13.5-17.5 g/dL (male)
Hct	37%	41-53% (male)
PLT	258 ×10⁹/L	150-400 ×10⁹/L
TP	6.3 g/dL	6.0-8.3 g/dL
LDH	240 U/L	140-280 U/L
ALP	200 U/L	44-147 U/L
ALT	10 U/L	7-56 U/L
AST	9 U/L	10-40 U/L
TB	0.5 mg/dL	0.1-1.2 mg/dL
BUN	20 mg/dL	7-20 mg/dL
Cr	1.2 mg/dL	0.7-1.3 mg/dL (male)
Na	136 mmol/L	135-145 mmol/L
K	4.5 mmol/L	3.5-5.0 mmol/L
Ca	8.5 mg/dL	8.6-10.2 mg/dL
Mg	1.5 mg/dL	1.7-2.2 mg/dL
Albumin	3 g/dL	3.5-5.0 g/dL
Cholesterol	250 mg/dL	<200 mg/dL
HDL	35 mg/dL	>40 mg/dL (male)
TG	170 mg/dL	<150 mg/dL
Glucose (blood)	110 mg/dL	70-99 mg/dL (fasting)
CRP	>190 mg/L	<10 mg/L
PCT	0.3 ng/mL	<0.05 ng/mL
Lactic acid	0.1 mmol/L	0.5-2.2 mmol/L
ABG pH	7.47	7.35-7.45
ABG PCO₂	35 mmHg	35-45 mmHg
ABG PO₂	45 mmHg	75-100 mmHg
ABG HCO₃	20 mmol/L	22-26 mmol/L
Pleural fluid WBC	500 /µL	<500 /µL (non-infectious)
Pleural fluid LDH	190 U/L	<200 U/L (transudate)
Pleural fluid TP	4 g/dL	<3 g/dL (transudate)
Pleural fluid glucose	50 mg/dL	>60 mg/dL

**Figure 1:**
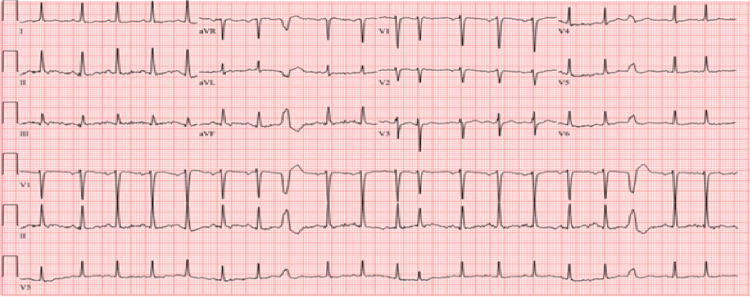
Electrocardiogram showing sinus tachycardia

The electrocardiogram showed sinus tachycardia (Figure 1).

**Figure 2:**
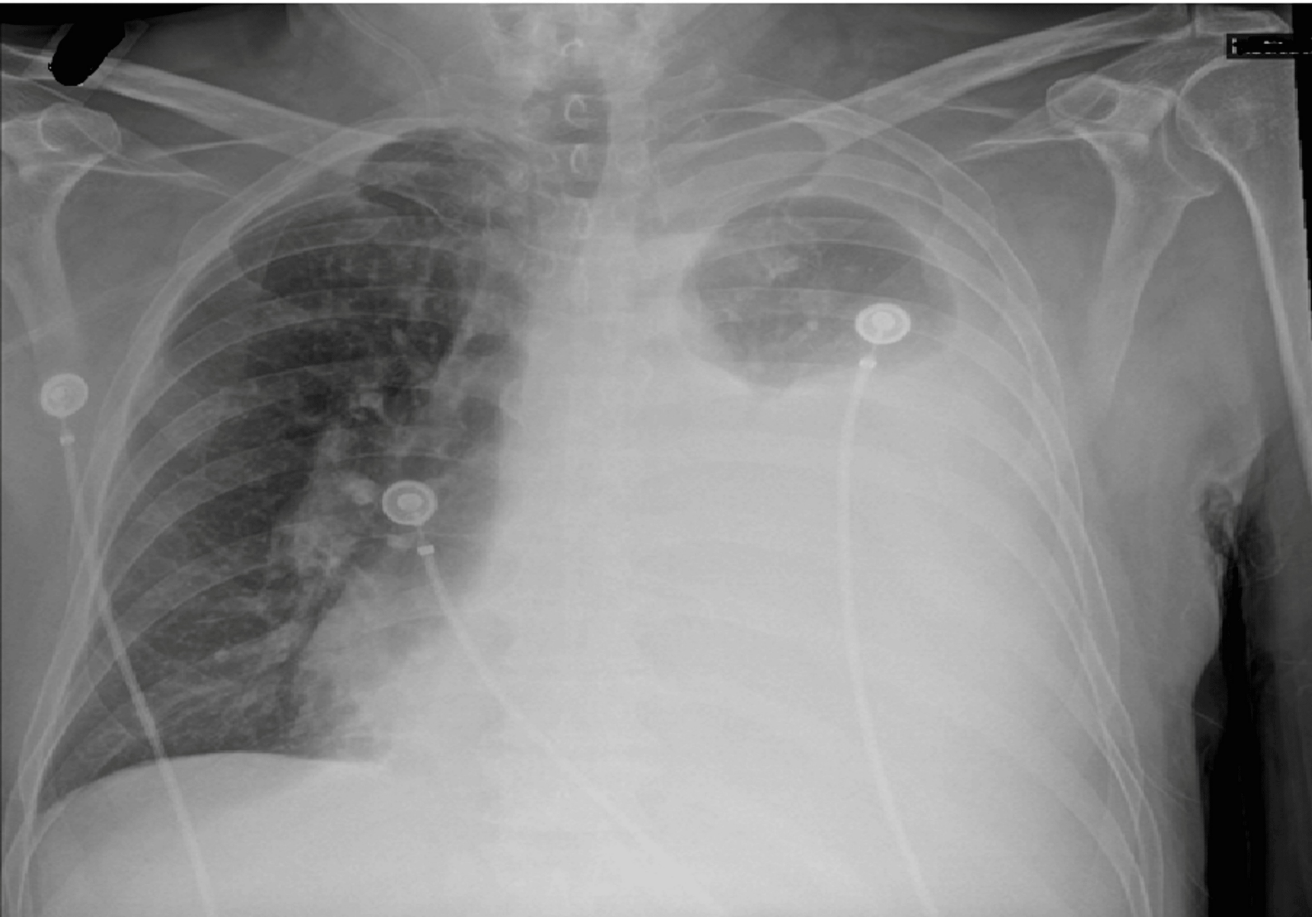
Chest X-ray Large left pleural effusion with minimal aeration of the left upper lobe.

Chest radiography revealed bilateral pleural effusions, more prominent on the left, with associated left lung atelectasis (Figure 2).

**Figure 3:**
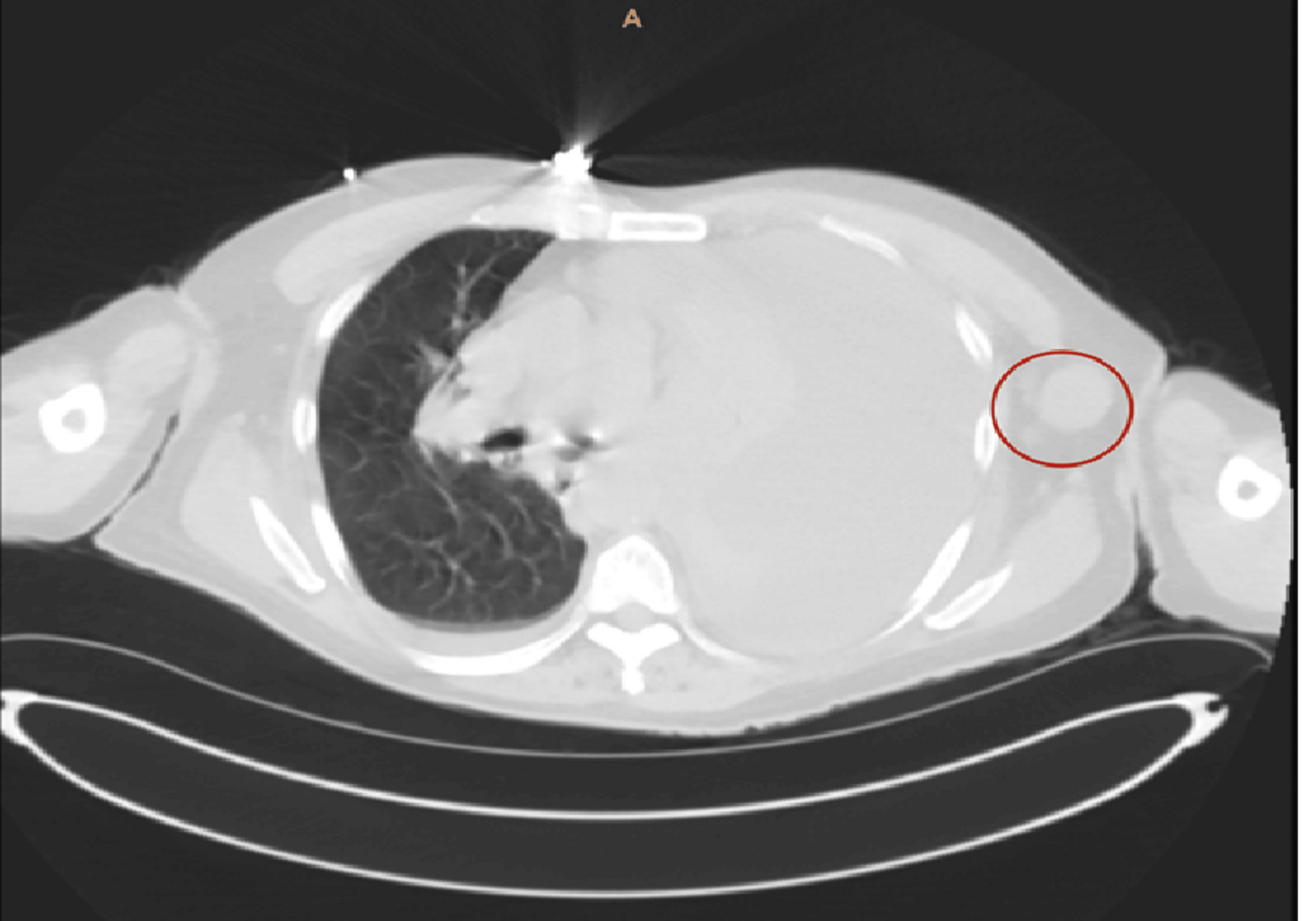
CT chest without contrast Prominent bilateral axillary lymph nodes, more pronounced on the left. A representative left axillary lymph node measures 2.3 cm in diameter (red circle). A left pleural effusion is also noted. CT: computed tomography

A CT of the chest, performed with and without contrast, demonstrated a large left‑sided pleural effusion, narrowing of the left mainstem bronchus, and mediastinal as well as axillary lymphadenopathy concerning for malignancy. The neck slices revealed no evidence of metastasis involving the cervical region or thyroid gland (Figure 3).

**Figure 4:**
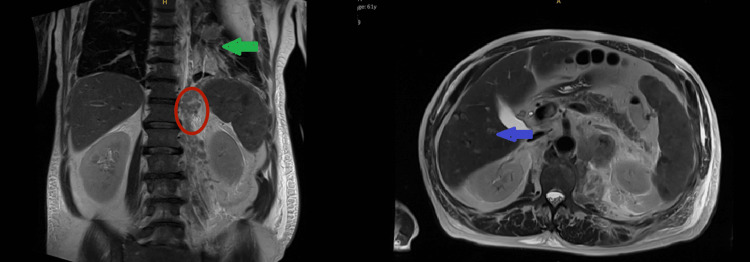
MRI of the abdomen without contrast Tiny scattered foci throughout the liver, with the largest measuring 15 mm in the right hepatic lobe (blue arrow). Adrenal nodules are present (red circle). A lung lesion is also noted (green arrow). MRI: magnetic resonance imaging

Further staging with abdominal MRI demonstrated splenomegaly with innumerable foci of restricted diffusion and hypoenhancement suggestive of malignant infiltration, a left adrenal mass consistent with metastasis, and an infiltrative lesion involving the left pleural space with transpleural extension. Multiple hepatic lesions were noted, the largest measuring 15 mm in the right hepatic lobe (Figure 4).

Initial management included supplemental oxygen via nasal cannula (2 L/min), bronchodilator therapy (albuterol/ipratropium), oral prednisone, azithromycin, and guideline-directed medical therapy for heart failure. A left-sided chest tube was placed, draining 1.5 liters of pleural fluid over two days. Pleural fluid analysis revealed an exudative effusion with negative cultures and cytology on two separate samples.

**Figure 5:**
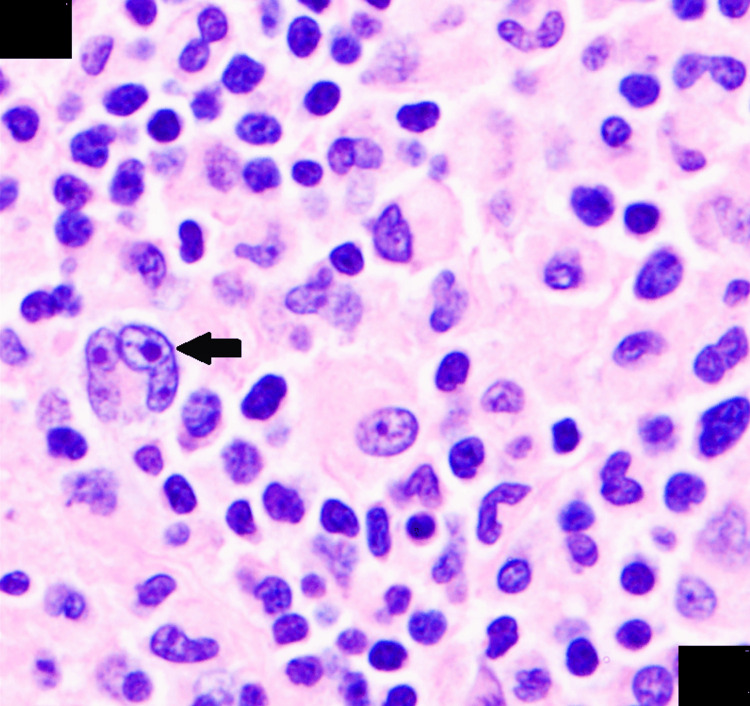
Lymph node biopsy Histopathological examination of lymph node tissue demonstrates classic features of HL. Large binucleated Reed-Sternberg cells with prominent eosinophilic nucleoli are observed (black arrow) within a mixed inflammatory background composed predominantly of small lymphocytes. Hematoxylin and eosin stain; magnification 100×, oil immersion objective. HL: Hodgkin lymphoma

A lymph node biopsy from the left axilla confirmed cHL, lymphocyte-rich subtype. Immunohistochemistry demonstrated positivity for CD30, CD15, PAX5, Ki-67, MOM1, and BCL6, with negative staining for CD20, CD3, LCA, and CD23. EBER-ISH highlighted abundant large atypical lymphocytes consistent with Hodgkin cells (Figure 5). Due to rapid reaccumulation of pleural fluid, a PleurX catheter was placed for ongoing drainage.

A nuclear medicine bone scan showed no evidence of osseous metastasis. During the initial days of hospitalization, the patient developed intermittent fever spikes with rising PCT levels (up to 3.5 ng/mL) and declining serum albumin. Despite an extensive infectious workup, including blood, sputum, and urine cultures; CT imaging of the chest, abdomen, and pelvis; pulmonary viral and bacterial PCR panels; and a nuclear medicine white blood cell tag scan, all results were negative. Empiric broad-spectrum antimicrobial therapy was initiated (vancomycin, piperacillin-tazobactam, metronidazole, and fluconazole), yet PCT continued to rise (peak 4.5 ng/mL).

On hospital day ten, the patient developed hypotension requiring transfer to the intensive care unit, where he subsequently died. The new onset of shock was likely multifactorial, attributable to the advanced stage of HL, underlying heart failure with reduced ejection fraction, severe pleural effusion necessitating drainage every three days via a PleurX catheter, and a possible infectious process. Despite extensive evaluation, including urinalysis, blood cultures, chest radiography, and repeated tagged white blood cell scans, no infectious source was identified. A comprehensive summary of the laboratory and diagnostic workup at the time of shock is provided in Table 2.

**Table 2:** Comprehensive summary of the laboratory and diagnostic workup at the time of shock WBC: white blood cell count, Hg: hemoglobin, Hct: hematocrit, PLT: platelets, TP: total protein, LDH: lactate dehydrogenase, ALP: alkaline phosphatase, ALT: alanine aminotransferase, AST: aspartate aminotransferase, TB: total bilirubin, BUN: blood urea nitrogen, Cr: creatinine, Na: sodium, K: potassium, Ca: calcium, Mg: magnesium, CRP: C-reactive protein, PCT: procalcitonin, IL-6: interleukin-6

Parameter	Patient value	Reference range
WBC	11 ×10⁹/L	4.0-11.0 ×10⁹/L
Hg	10.5 g/dL	13.5-17.5 g/dL (male)
Hct	36%	41-53% (male)
PLT	233 ×10⁹/L	150-400 ×10⁹/L
TP	7 g/dL	6.0-8.3 g/dL
LDH	270 U/L	140-280 U/L
ALP	210 U/L	44-147 U/L
ALT	25 U/L	7-56 U/L
AST	35 U/L	10-40 U/L
TB	1 mg/dL	0.1-1.2 mg/dL
BUN	29 mg/dL	7-20 mg/dL
Cr	1.4 mg/dL	0.7-1.3 mg/dL (male)
Na	140 mmol/L	135-145 mmol/L
K	4 mmol/L	3.5-5.0 mmol/L
Ca	8 mg/dL	8.6-10.2 mg/dL
Mg	1.2 mg/dL	1.7-2.2 mg/dL
Albumin	2.9 g/dL	3.5-5.0 g/dL
Glucose	100 mg/dL	70-99 mg/dL (fasting)
CRP	>190 mg/L	<10 mg/L
PCT	4.5 ng/mL	<0.05 ng/mL
Lactic acid	2.1 mmol/L	0.5-2.2 mmol/L
IL-6	39 pg/mL	0-5 pg/mL
Pleural fluid WBC	750 /µL	<500 /µL (non-infectious)
Pleural fluid LDH	200 U/L	<200 U/L (transudate)
Pleural fluid TP	3.5 g/dL	<3 g/dL (transudate)
Pleural fluid glucose	60 mg/dL	>60 mg/dL

## Discussion

cHL accounts for approximately 85-95% of all HL cases [6,7]. Most patients with cHL present with lymphadenopathy. The commonly involved nodal sites include cervical, mediastinal, supraclavicular, and axillary, with some variation in site preference among different subtypes. Extranodal involvement usually arises from hematogenous dissemination, while primary extranodal disease is rare. The most commonly involved extranodal sites include the lungs, liver, and bone. cHL exhibits a bimodal age distribution with one peak in the pediatric age group and a second peak over 60 years [7].

Staging of HL is primarily determined through imaging modalities such as PET and CT, which provide critical anatomical and metabolic information. According to the Ann Arbor classification, stage I disease involves a single lymph node region or lymphoid organ (I) or a solitary extranodal site (IE). Stage II is defined by involvement of two or more lymph node regions on the same side of the diaphragm (II) or localized extranodal extension with adjacent nodal involvement (IIE). Stage III indicates lymph node involvement on both sides of the diaphragm, potentially including the spleen. Stage IV reflects disseminated disease with one or more extranodal organs affected, such as the bone marrow, liver, or lungs, with or without concurrent lymph node involvement [8]. For stage 4 HL, the chance of surviving at least five years is about 82% [9].

The patient, a 61-year-old individual, presented with clinical symptoms suggestive of lymphoproliferative disease. A diagnosis of cHL was established through axillary lymph node biopsy and flow cytometric analysis, which demonstrated B lymphocytes expressing CD30, CD15, PAX5, Ki-67, MUM1, and BCL markers. Staging CT revealed hepatic and splenic involvement, consistent with stage IV disease according to the Ann Arbor classification.

Multiple prognostic factors have been investigated in HL, with age representing a key determinant due to its association with comorbidities and decreased tolerance to chemotherapy in older patients compared to younger cohorts. Gender also influences outcomes, as males typically exhibit a poorer prognosis than females [4]. Hypoalbuminemia has been consistently associated with unfavorable clinical outcomes, a relationship that may be mechanistically explained by the suppression of hepatic albumin synthesis during inflammatory states. Specifically, the upregulation of acute-phase protein production mediated by IL-6 and the reduced availability of amino acids secondary to compromised nutritional status contribute to diminished albumin synthesis. Moreover, circulating albumin concentrations are inversely correlated with key inflammatory mediators, including IL-6, TNF-α, and IL-1RA [4,10]. In the present case, the male patient’s advanced age, concomitant heart failure with reduced ejection fraction, low serum albumin levels, and stage IV cHL likely contribute collectively to a less favorable prognosis.

Markers of systemic inflammation are frequently elevated in patients with HL, reflecting disease activity and offering potential utility as diagnostic and prognostic indicators. At the time of diagnosis, approximately 70% of HL cases exhibit elevated CRP, neutrophilic leukocytosis, and increased serum ferritin levels, while PCT typically remains within normal limits [11,12].

However, emerging evidence suggests that PCT may be elevated in more advanced stages of HL. In a study by Piperidou et al. [12], 137 newly diagnosed HL patients were evaluated, including 79 with advanced-stage disease. The authors reported that PCT levels exceeded 0.1 ng/mL in patients with advanced disease, with two cases exceeding 0.5 ng/mL (p<0.001). These findings suggest that PCT elevation may be linked to the severity of the inflammatory response in HL, potentially driven by elevated pro-inflammatory cytokines such as TNF-α and IL-6.

Although PCT is classically elevated in bacterial infections, patients in the study had negative infectious workups (blood, sputum, and urine cultures; CT imaging of the chest, abdomen, and pelvis; pulmonary viral and bacterial PCR panels; and nuclear medicine white blood cell tag scan) and were treated empirically with broad-spectrum antibiotics and antifungals without subsequent decline in PCT levels (patient’s PCT trended up to 5 ng/ml despite antibiotic and antifungal coverage). This observation supports the hypothesis that PCT elevation in HL may be independent of infection and instead reflects disease-related inflammation and prognosis.

## Conclusions

These findings suggest that PCT may serve as a supplementary biomarker in HL, particularly in advanced stages of disease. Its elevation could aid prognostication and risk stratification, warranting further investigation in larger prospective cohorts. However, further research is warranted to validate this potential correlation and strengthen its role in clinical decision-making.
